# Ubiquitin-Specific Protease 25 Aggravates Acute Pancreatitis and Acute Pancreatitis-Related Multiple Organ Injury by Destroying Tight Junctions Through Activation of The STAT3 Pathway

**DOI:** 10.3389/fcell.2021.806850

**Published:** 2022-01-13

**Authors:** Zhengru Liu, Mingming Qi, Shan Tian, Qian Yang, Jian Liu, Siwei Wang, Mengyao Ji, Rong Yu, Suqi Zeng, Jinting Li, Yuping Wei, Weiguo Dong

**Affiliations:** Department of Gastroenterology, Renmin Hospital of Wuhan University, Wuhan, China

**Keywords:** acute pancreatitis, ubiquitin-specific protease 25, multiple organ injury, tight junctions, signal transducer and activator of transcription 3 pathway

## Abstract

Ubiquitin-specific protease 25 (USP25) plays an important role in inflammation and immunity. However, the role of USP25 in acute pancreatitis (AP) is still unclear. To evaluate the role of USP25 in AP, we conducted research on clinical AP patients, USP25^wild-type(WT)^/USP25 knockout (USP25^−/−^) mice, and pancreatic acinar cells. Our results showed that serum USP25 concentration was higher in AP patients than in healthy controls and was positively correlated with disease severity. AP patients’ serum USP25 levels after treatment were significantly lower than that at the onset of AP. Moreover, USP25 expression was upregulated in cerulein-induced AP in mice, while USP25 deficiency attenuates AP and AP-related multiple organ injury. *In vivo* and *in vitro* studies showed that USP25 exacerbates AP by promoting the release of pro-inflammatory factors and destroying tight junctions of the pancreas. We showed that USP25 aggravates AP and AP-related multiple organ injury by activating the signal transducer and activator of transcription 3 (STAT3) pathway. Targeting the action of USP25 may present a potential therapeutic option for treating AP.

## Introduction

Acute pancreatitis (AP) is characterized by autodigestion, edema, hemorrhage, and even necrosis of the pancreatic tissue after activation of pancreatin. Inflammation of the pancreas with or without organ failure can be observed during the course of pancreatitis. AP can be classified according to severity into mild acute pancreatitis (MAP), moderate severe acute pancreatitis (MSAP), and severe acute pancreatitis (SAP) (Crockett et al., 2018). Approximately 20% of AP patients develop SAP, which is related to systemic inflammatory response and multiple organ injury. Lung, liver, kidney, and heart are organs that are often involved in AP-related multiple organ failure (Schepers et al., 2019). According to global estimates, the incidence of acute pancreatitis is 34 cases per 100,000 persons each year with a 95% confidence interval [23.33, 48.81], and the mortality rate is 1.60 cases per 100,000 persons each year with a 95% confidence interval [0.85, 1.58] (Xiao et al., 2016). With increase in the global obesity rate, obesity-related complications, such as cholelithiasis, hypertriglyceridemia, and diabetes, are independently related to the onset of AP (Black and Gupta, 2018). The increasing incidence of AP has brought a heavy economic burden to global medical care. However, there is still no effective method to prevent AP.

The intercellular spaces between pancreatic epithelial cells are usually closed by tight junctions. These connections are assembled from the components of adjacent cells that form a gasket-like band that surrounds the surface of each cell at the intersection of the apical and basolateral regions. This seal restricts the movement of molecules between the epithelial cavity and the interstitium according to molecule charge and size. Structurally, tight junctions are related to the actin-based cytoskeleton (Rouaud et al., 2020). Several tight junction-related proteins have been identified, and zonula occludens (ZO)-1 is the most widely used structural marker for tight junctions. It has been shown that the expression of ZO-1 is downregulated in AP and the expression of ZO-1 is negatively correlated with the degree of inflammation (Sharmila and Venkateswaran, 2017).

Deubiquitinating enzymes (DUBs) inversely regulate protein ubiquitination by dissociating ubiquitin chain molecules and maintaining the dynamic balance of cell ubiquitin. DUBs play an important role in the regulation of protein degradation and ubiquitin recovery, DNA repair pathways, and cell signal regulation (Clague et al., 2019). DUBs can be classified into six subfamilies: ubiquitin-specific proteases (USPs), ovarian tumor proteases, ubiquitin carboxy-terminal hydrolases, Josephine family, ubiquitin-interacting enzymes motif, zinc-dependent JAB1/MPN/MOV34 metalloprotease DUBs (JaMs) (Mevissen and Komander, 2017).

Ubiquitin-specific protease 25 (USP25) belongs to the cysteine protease family of DUBs, and is involved in the regulation of inflammation and immunity. Long et al. found that USP25 can promote the stability of histone acetyltransferase HBO1 in bacterial infections, thereby enhancing HBO1-mediated transcription of inflammatory genes and promoting inflammation (Long et al., 2018). USP25 is also involved in the immune regulation of colon tumors and intestinal bacterial infections by regulating the levels of Wnt and suppressor of cytokine signaling 3 (SOCS3) (Wang et al., 2020). In addition, USP25 is upregulated after viral infection and participates in the body’s antiviral response by mediating the stabilization of tumor necrosis factor (TNF) receptor associated factor 3 (TRAF3) and TRAF6 (Lin et al., 2015). However, the role of USP25 and its mechanism in acute pancreatitis remains unclear.

Here, we investigated whether USP25 affects inflammation in patients with AP. We found that serum USP25 concentrations were higher in AP patients than in healthy controls and were positively correlated with disease severity in AP patients. We demonstrated that USP25 plays a critical role in mediating AP development by activating the STAT3 pathway.

## Materials and Methods

### Serum Collection

Human serum samples were collected at the Renmin Hospital of Wuhan University, and informed consent was obtained from all patients and healthy controls. The study was approved by the Ethics Committee of the Renmin Hospital of Wuhan University (No. 2018 K-C089). The diagnosis of AP was based on *the Atlanta classification of acute pancreatitis (2012 revision)* (Banks et al., 2013). Patients’ clinical information were collected; this includes information regarding sex, age, and blood examination results, including serum amylase (AMY), lipase (LIPA), alanine aminotransferase (ALT), aspartate aminotransferase (AST), creatinine (Cr), blood urea nitrogen (BUN), and C-reactive protein (CRP). We then used the human USP25 enzyme-linked immunosorbent assay (ELISA) kit (Meilian ml-895378, Suzhou, China) to measure the patients’ serum USP25 protein levels according to the manufacturer’s instructions. Serum interleukin (IL)-6, TNF-α, and IL-1β levels were examined using ELISA kits (Quantikine; R&D Systems, United States). The clinical characteristics of the patients are summarized in Table 1. We followed up with 16 patients who were discharged from the hospital after treatment and examined the serum USP25 protein levels on admission and after treatment.

**TABLE 1:** Clinical characteristics of patients.

	Control	AP group	*p*-value
Number	53	79	
Gender	23/30	38/41	0.4572
Age	52.49 ± 10.67	51.57 ± 11.78	0.6505
AMY (U/L)	61.47 ± 12.94	481.10 ± 75.59	<0.0001
LIPA (U/L)	58.42 ± 13.26	1870.54 ± 554.13	<0.0001
ALT (U/L)	27.28 ± 7.25	98.23 ± 31.93	0.0154
AST (U/L)	24.43 ± 5.42	59.57 ± 22.28	0.0245
Cr (μmol/L)	59.83 ± 11.61	110.53 ± 38.64	<0.0001
BUN (mmol/L)	5.26 ± 1.18	15.88 ± 11.14	0.0041
CRP (mg/L)	4.98 ± 2.22	102.00 ± 93.90	0.0017
IL-6 (pg/ml)	67.79 ± 8.73	233.76 ± 124.09	<0.0001
TNF-α (pg/ml)	157.25 ± 31.10	1723.08 ± 738.01	<0.0001
IL-1β (pg/ml)	157.91 ± 62.86	1,259.58 ± 664.63	<0.0001

Abbreviation: AMY, amylase; LIPA, lipase; ALT, alanine amiotransferase; AST, aspartate amiotransferase; Cr, creatinine; BUN, blood urea nitrogen; CRP, C-creative protein; IL, interleukin; TNF, tumor necrosis factor.

### Induction of AP

Animal protocols were approved by the Animal Ethics Committee of Wuhan University for animal experiments (No. WDRY2018-K033). C57BL/6 USP25^−/−^ mice were kindly provided by Dr. Chen Dong (Institute for Immunology and School of Medicine, Tsinghua University, Beijing, China), and C57BL/6 wild-type (WT) mice were purchased from the Vital River Laboratory (Beijing, China). All experimental mice were housed in a specific pathogen-free animal facility at the Animal Experiment Center at Renmin Hospital of Wuhan University.

A 2% agarose gel electrophoresis was used to verify the DNA of USP25^WT^ and USP25^−/−^ mice. C57BL/6 USP25^WT^ and USP25^−/−^ male mice (6–8 weeks, 20 ± 0.8 g, n = 8 per group) were randomly divided into four groups: USP25^WT^ + normal saline (NS), USP25^WT^ + cerulein, USP25^−/−^ + NS, and USP25^−/−^ + cerulein. We administered intraperitoneal injections of cerulein (Sigma-Aldrich C9026, St. Louis, MO) (50 μg/kg body weight) seven times at an interval of 1 h to induce AP in mice (Silva-Vaz et al., 2019); the control group was administered 0.9% NS. Twenty-four hours after the last injection of cerulein and NS, mouse blood was collected from the inferior vena cava after the mice were anesthetized with isoflurane. The mice were sacrificed, and the pancreas, lungs, livers, kidneys, and small intestines of mice were immediately removed.

To induce AP *in vitro*, rat pancreatic acinar cells (AR42J) and mouse pancreatic acinar cells (MPAC) were treated with 10^-8^ M cerulein, and the control group was treated with the same amount of phosphate buffered saline (PBS).

### Inhibition of STAT3 Activation With Stattic

Stattic (ab120952, Abcam, Cambridge, UK) was administered to mice at a dose of 1 mg/kg/day per mouse for 7 days to inhibit STAT3 phosphorylation, and then mice were injected intraperitoneally with NS or cerulein. In the *in vitro* study, the cells AR42J and MPAC were incubated with 10 μM stattic for 2 h, followed by the addition of PBS or cerulein.

### Activation of STAT3 Phosphorylation With Colivelin

In the colivelin treatment study, the cells AR42J and MPAC were incubated with 100 fM colivelin, followed by the addition of cerulein.

### Serum Examination

The blood samples were centrifuged at 3,000 × *g* at 4°C for 10 min, and the serum was stored at −80°C before examination. Serum AMY, LIPA, ALT, AST, Cr, and BUN were measured using a fully automated chemical analyzer (Chemray 800; Rayto Life Technology Co., Ltd. Shengzhen, China). Serum IL-6, TNF-α, and IL-1β levels were examined using ELISA kits (Quantikine; R&D Systems, United States) according to the manufacturer’s instructions. All samples were analyzed three times.

### Histological Examination

The pancreases, lungs, livers, kidneys, and small intestines obtained from the experimental mice were fixed in 4% buffered formalin for 24 h and then embedded in paraffin. Tissue sections (4 μm) were stained with hematoxylin and eosin (H&E). The histopathological changes were analyzed under an optical microscope by two expert pathologists who were blinded to the experimental method.

The pathological changes in pancreatic tissue were evaluated according to the criteria proposed by Schmidt et al., including edema, inflammation, perivascular infiltration, hemorrhage, fat necrosis, and acinar necrosis (Schmidt et al., 1992). Lung injury assessment was based on the criteria described by Osman et al., including interstitial and alveolar edema, leukocyte infiltration, and hemorrhage (Osman et al., 1998). The evaluation of liver damage was described by Moto et al., including hepatocyte necrosis, leukocyte infiltration, and vacuolation (Mota et al., 2007). The pathological changes of renal injury were evaluated according to the criteria proposed by Paller et al., examining at least 100 cortical tubules in ten different areas for renal tubular epithelial cell flattening, brush border deletion, cell membrane bubble formation, interstitial edema, cytoplasmic vacuoles, cell necrosis, and renal tubule obstruction (Paller et al., 1984). The evaluation of small intestine injury was based on the standard method proposed by Chiu et al., including the distance of the small intestine villi and the detachment of the villi from the lamina propria (Chiu et al., 1970).

### Immunohistochemistry

After deparaffinization, hydration, antigen retrieval, and serum block, pancreatic sections were incubated overnight with the following primary antibodies at 4°C: CD45 (1:1,000, catalog no.20103-1-AP, Proteintech, Wuhan, China) and USP25 (1:200, catalog no. A7975, ABclonal). Goat anti-rabbit horseradish peroxidase (1:200; catalog no. AS-1107, Aspen, Wuhan, China), or goat anti-mouse horseradish peroxidase (1:200; catalog no. AS-1106, Aspen Wuhan, China) was added to the slices.

### Immunofluorescence

After the paraffin sections of the pancreas were deparaffinized, washed, and blocked, ZO-1 antibody (1:1,000; catalog no. 21773-1-AP, Proteintech, Wuhan, China) was added to it and incubated at 4°C. MPAC were incubated with p-STAT3^Y705^ antibody (1:100; catalog no. #9145, Cell Signaling Technology) at 4°C. After washing with PBS, CY3-labeled goat anti-rabbit antibody (1:50; catalog no. AS-1109, Aspen, Wuhan, China) was added, and the nuclei were stained with 4,6-diamino-2-phenylindole (DAPI, AS1075, Aspen, Wuhan, China). To evaluate the expression level of ZO-1 and p-STAT3, ImageJ (National Institutes of Health, Bethesda, MD, USA) was used to measure the fluorescence intensity of ZO-1 and p-STAT3, and the fluorescence intensity was quantified under a high-power field.

### Western Blotting

A total protein extraction kit (Beyotime Bio-technology) was used to extract total protein from different groups, and protein concentration was determined using the BCA kit (P0012, Beyotime Bio-technology, Shanghai, China). From each group, a 20 μg protein sample was electrophoresed on a 10% SDS polyacrylamide gel and then transferred to a polyvinylidene fluoride membrane. After blocking the protein with a protein-free rapid blocking buffer (catalog no. PS108P, EpiZyme, Shanghai, China), polyvinylidene fluoride membranes were incubated for 12 h at 4°C with the following antibodies: USP25 antibody (1: 1,000; catalog no. A7975, ABclonal), STAT3 antibodies (1:1,000; catalog no. #9139, Cell Signaling Technology), p-STAT3 (Tyr705) antibody (1:2,000; catalog no. #9145, Cell Signaling Technology), ZO-1 antibody (1:1,000; catalog no. 21773-1-AP, Proteintech, Wuhan, China), and glyceraldehyde 3-phosphate dehydrogenase (GAPDH) antibody (1:1,000; catalog no. #5174, Cell Signaling Technology).

### Cell Culture

Mouse primary acinar cells were cultured as described below. Briefly, pancreas were isolated from 8-week-old male USP25 KO and WT C57BL/6J mice and minced for 5 min in Hank’s.

Balanced salt solution (Sigma, St. Louis, MO, USA) + 10 mmol/L of HEPES (Sigma, St. Louis, MO, United States) and 0.1% bovine serum albumin (Gibco, Grand Island, NY, United States). After washing, pancreatic segments were cultured in 10 ml collagenasa IA solution (Hank’s balanced salt solution Contains 10 mmol/L HEPES, 0.25 mg/ml trypsin inhibitor and 200 U/mL collagenase IA [Sigma, St. Louis, MO, United States]) at 37°C for 20-30 min. Then the solution containing collagenase was removed and replaced with Dulbecco’s modified Eagle medium (Sigma, St. Louis, MO, United States) supplemented with 10% fetal bovine serum (Gibco, Grand Island, NY, United States), 1% Penicillin and streptomycin (Invitrogen, Carlsbad, CA, United States), 25 ng/ml recombinant mouse epidermal growth factor (Sigma, St. Louis, MO, United States) and 0.25 mg/ml trypsin inhibitor at 37°C, 95% humidity and 5% CO_2_.

AR42J cells were purchased from the American Type Culture Collection (Manassas, VA, United States). The cells were cultured in a 5% CO2 humidified incubator at 37°C in Dulbecco’s modified Eagle’s medium (Sigma, St. Louis, MO, United States) supplemented with 10% fetal bovine serum (Gibco, Grand Island, NY, United States), 1% Penicillin and streptomycin (Invitrogen, Carlsbad, CA, United States).

### Cell Transfection

Small interfering RNA (siRNA) targeting the *Usp25* gene (siRNA *Usp25*) and non-targeting siRNAs (NC) were purchased from GenePharma Genomics Co. Ltd. (Suzhou, China). Cells were cultured with Lipofectamine 6000 (Beyotime, China) and transfected with siRNA according to the manufacturer’s instructions. After the cells were incubated with siRNA for 72 h, cell protein was extracted and transfection efficiency was verified by WB.

### Statistical Analysis

Statistical analyses were performed using the GraphPad Prism software version 8.0. Data are expressed as mean ± SD. Comparisons between the two groups were performed using the unpaired Student’s t-test for normally distributed parameters and the Wilcoxon rank-sum test for non-normally distributed parameters. The Pearson correlation analysis was used to test correlations, and one-way analysis of variance (ANOVA) combined with the Bonferroni’s post hoc test was used for multi-group testing. Statistical significance was defined at *p* < 0.05.

## Results

### Serum USP25 Concentration is Higher in Patients With AP Than in Healthy Controls and Positively Correlated With the Disease Severity and Multiple Organ Injury

**FIGURE 1:**
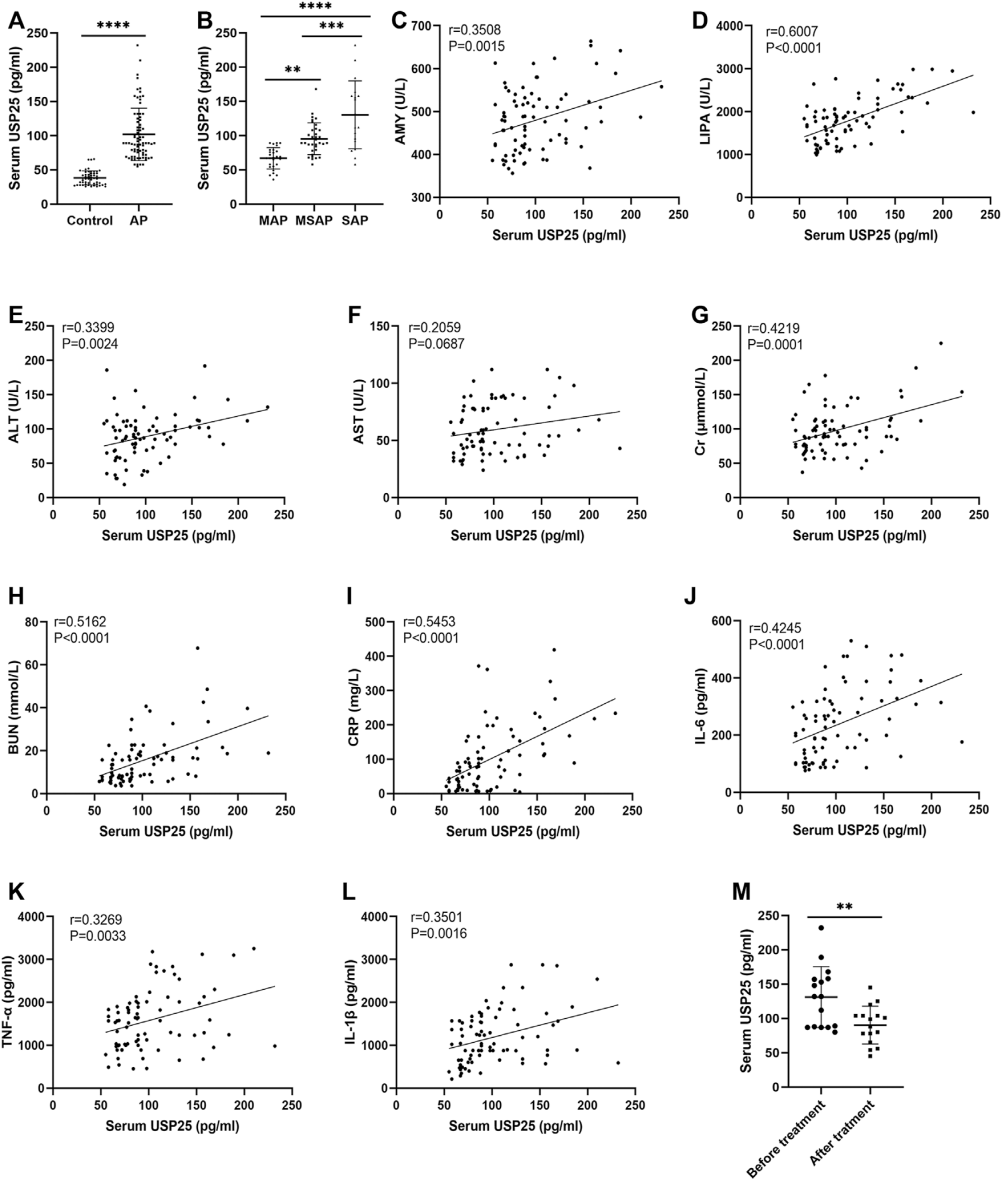
Serum USP25 concentrations is higher in patients with acute pancreatitis and positively correlated with the disease severity and multiple organ injury. **(A)** USP25 expression in serum samples from healthy controls (n = 53) and acute pancreatitis (AP) patients (n = 79) was detected by ELISA. **(B)** Serum USP25 concentrations in MAP (n = 27), MSAP (n = 34), and SAP (n = 18) patients were compared. **(C–L)** The relationship between serum USP25 and AMY, LIPA, ALT, AST, Cr, BUN, CRP, IL-6, TNF-α, and IL-1β (n = 79) were analyzed. **(M)** Serum USP25 concentrations of AP patients (n = 16) before and after treatment were compared. **p < 0.01, ***p < 0.001 and ****p < 0.0001 compared with control groups.

We collected clinical data from 79 patients diagnosed with AP (27 MAP patients, 34 MSAP patients, and 18 SAP patients) and 53 healthy controls, and serum USP25, IL-6, TNF-α, and IL-1β levels in AP patients and healthy controls were detected by ELISA. As shown in Table 1, compared with healthy individuals, the level of serum AMY, LIPA, ALT, AST, Cr, BUN, CRP, IL-6, TNF-α, and IL-1β increased significantly (*p* < 0.05). The levels of serum USP25 protein in patients and controls from ELISA are shown in Figure 1A. The serum USP25 levels of AP patients were significantly higher than that of the healthy controls (38.36 ± 10.27 pg/ml vs 101.97 ± 61.25 pg/ml; *p* < 0.05). In addition, as shown in Figure 1B, SAP patients had the highest serum USP25 protein level (161.17 ± 27.58 pg/ml), followed by MSAP patients (97.24 ± 12.31 pg/ml), and MAP patients had the lowest USP25 protein level (68.48 ± 6.82 pg/ml).

In the correlation analysis between serum markers, and USP25 (Figures 1C–1L), AMY (r = 0.3508, *p* < 0.05), LIPA (r = 0.6007, *p* < 0.05), ALT (r = 0.3399, *p* < 0.05), Cr (r = 0.4219, *p* < 0.05), BUN (r = 0.5162, *p* < 0.05), CRP (r = 0.5453, *p* < 0.05), IL-6 (r = 0.4246, *p* < 0.05), TNF-α (r = 0.3269, *p* < 0.05), and IL-1β (r = 0.3501, *p* < 0.05) levels were positively related with the USP25 level. We followed up with 16 AP patients to compare the serum USP25 level before and after treatment (Figure 1M), and the patients’ serum USP25 levels were significantly lower after treatment than that at the onset of AP (131.19 ± 42.94 pg/ml vs 90.38 ± 26.73 pg/ml, *p* < 0.05).

### The Expression of USP25 Is Upregulated in Cerulein-Induced AP Mice and Associated With Multiple Organ Injury

**FIGURE 2:**
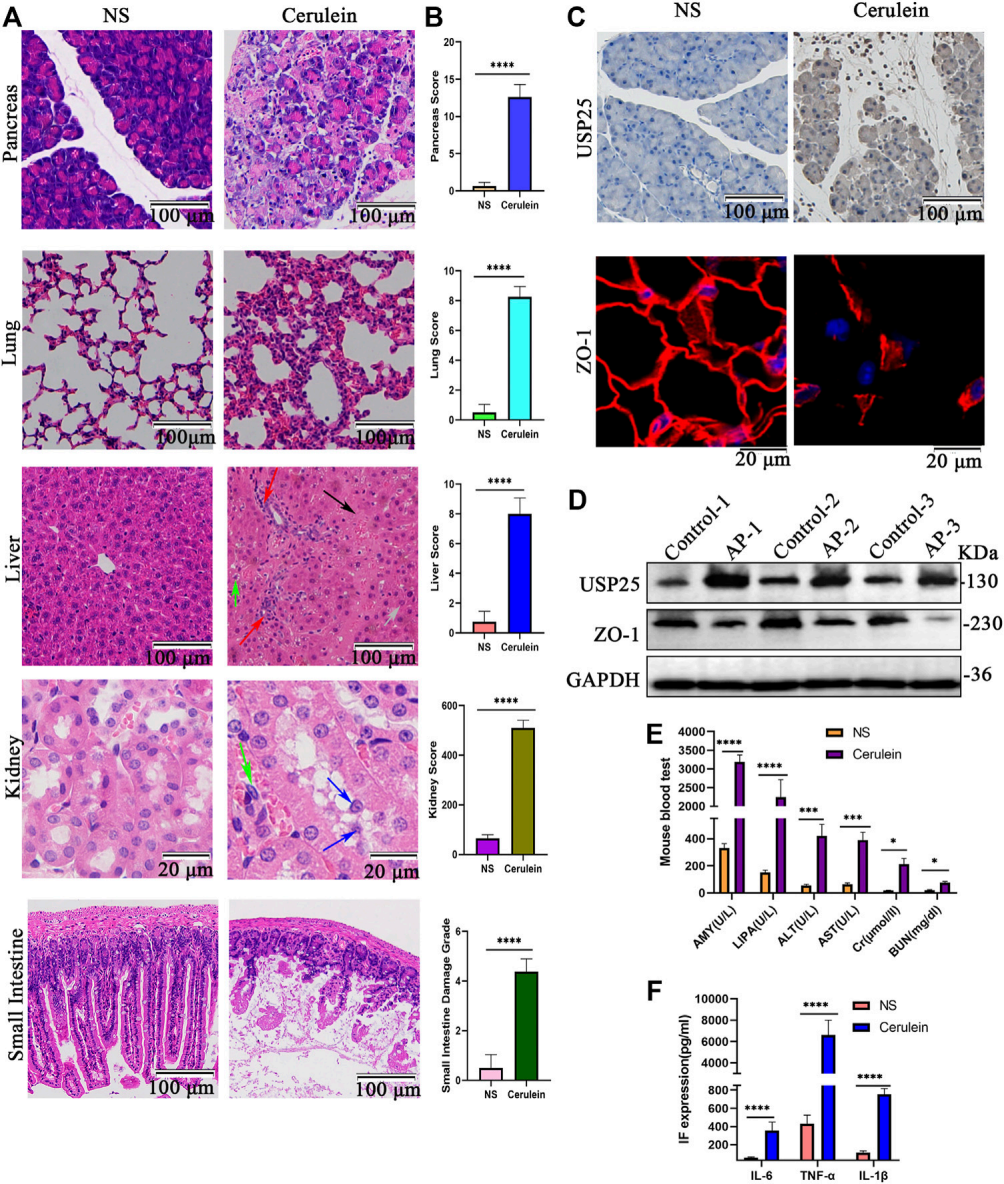
The expression of USP25 is up-regulated in cerulein-induced acute pancreatitis mice and associated with multiple organ injury. Wide type mice were randomly divided into two groups (n = 8 per group), control group: USP25^WT^ + normal saline (NS) and AP group: USP25^WT^ + cerulein. **(A)** H&E staining of pancreas, lung, liver, kidney, and small intestine in control mice and AP mice (Black arrow: necrosis; red arrow: inflammatory cell; green arrow: cytoplasmic vacuolation; grey arrow: edema; blue arrow: detachment of tubular epithelial cell). **(B)** The histological scores of pancreas, lung, liver, kidney, and small intestine in control mice and AP mice. **(C)** The protein expression level of USP25 and ZO-1 in control and AP mice pancreases were examined by immunohistochemistry and immunofluorescence respectively. **(D)** The USP25 protein level as well as ZO-1 was evaluated by Western blotting. **(E)** The levels of serum AMY, LIPA, ALT, AST, Cr, BUN were examined by automatic chemical analyzer in control and AP mice. **(F)** The levels of serum inflammatory factor (IF) IL-6, TNF-α, and IL-1β in control and AP mice were measured by ELISA. *p < 0.05, ***p < 0.001 and ****p < 0.0001 compared with control groups.

After multiple intraperitoneal injections of cerulein to induce AP in mice, mouse pancreas weight (g)/mouse weight (g) was calculated, and the mouse pancreas, lung, liver, kidney, and small intestine were examined using H&E staining. The results showed that the relative weight of the pancreas of AP mice was significantly higher than that of the control group (*p* < 0.05) (Supplementary Figure 2A). As shown in Figure 2A, the pancreas of AP mice showed severe edema, large acinar necrosis, inflammatory cell infiltration, and intrapancreatic hemorrhage. Histopathological examination of lung H&E of AP mice showed interstitial and intra-alveolar edema, inflammatory cell infiltration, and hemorrhage in the interstitium and alveoli. Obvious hepatocyte edema, cytoplasmic vacuolation, necrosis, loss of intercellular boundaries, and inflammatory cell infiltration were observed in the livers of AP mice. The kidneys of the AP group showed obvious pathological changes, including detachment of tubular epithelial cells, dilated renal tubules, disordered tubular structure and inflammatory cell infiltration around the renal tubules. In addition, the small intestines of the AP group showed widening of the subepithelial space at the top of the villi and peeling of the villi tip from the lamina propria or falling off in pieces. In contrast, the histology of the pancreas, lung, liver, kidney, and small intestine in the control group were normal (Figure 2A). The histological scores of different organs in the AP mice were significantly higher than those in the control group (*p* < 0.05) (Figure 2B).

To further verify the difference in inflammation between wild-type mice treated with saline and cerulein, we stained the tissues for a marker of inflammation (CD45) by IHC (Supplementary Figure 1A) and quantified (Supplementary Figures 1B–1F). The results showed that tissues of mice treated with cerulein had higher infiltration of inflammatory cells than that of mice treated with saline (*p* < 0.05).

The expression levels of USP25 in mouse pancreas were examined by IHC and WB. The results are shown in Figures 2C,D and Supplementary Figures 2B, 2C. The expression levels of USP25 protein (IHC, *p* < 0.05; WB, *p* < 0.05) in the pancreas of AP mice were significantly higher than those of the control group.

Examination of mouse serum AMY, LIPA, ALT, AST, Cr, and BUN levels using an automatic chemical analyzer showed that AMY (*p* < 0.05), LIPA (*p* < 0.05), ALT (*p* < 0.05), AST (*p* < 0.05), Cr (*p* < 0.05), and BUN (*p* < 0.05) of AP mice were significantly higher than those of the control group (Figure 2E). The examination of serum inflammatory factors by ELISA showed that the levels of serum IL-6 (*p* < 0.05), TNF-α (*p* < 0.05), and IL-1β (*p* < 0.05) were significantly higher in AP mice than in the control group (Figure 2F).

Therefore, USP25 is upregulated in cerulein-induced AP mice and is associated with inflammation. We speculated that USP25 may promote the inflammatory response in AP.

### USP25 Deficiency Attenuates Pancreatic Inflammation and AP-Related Multiple Organ Injury *in Vivo*

**FIGURE 3:**
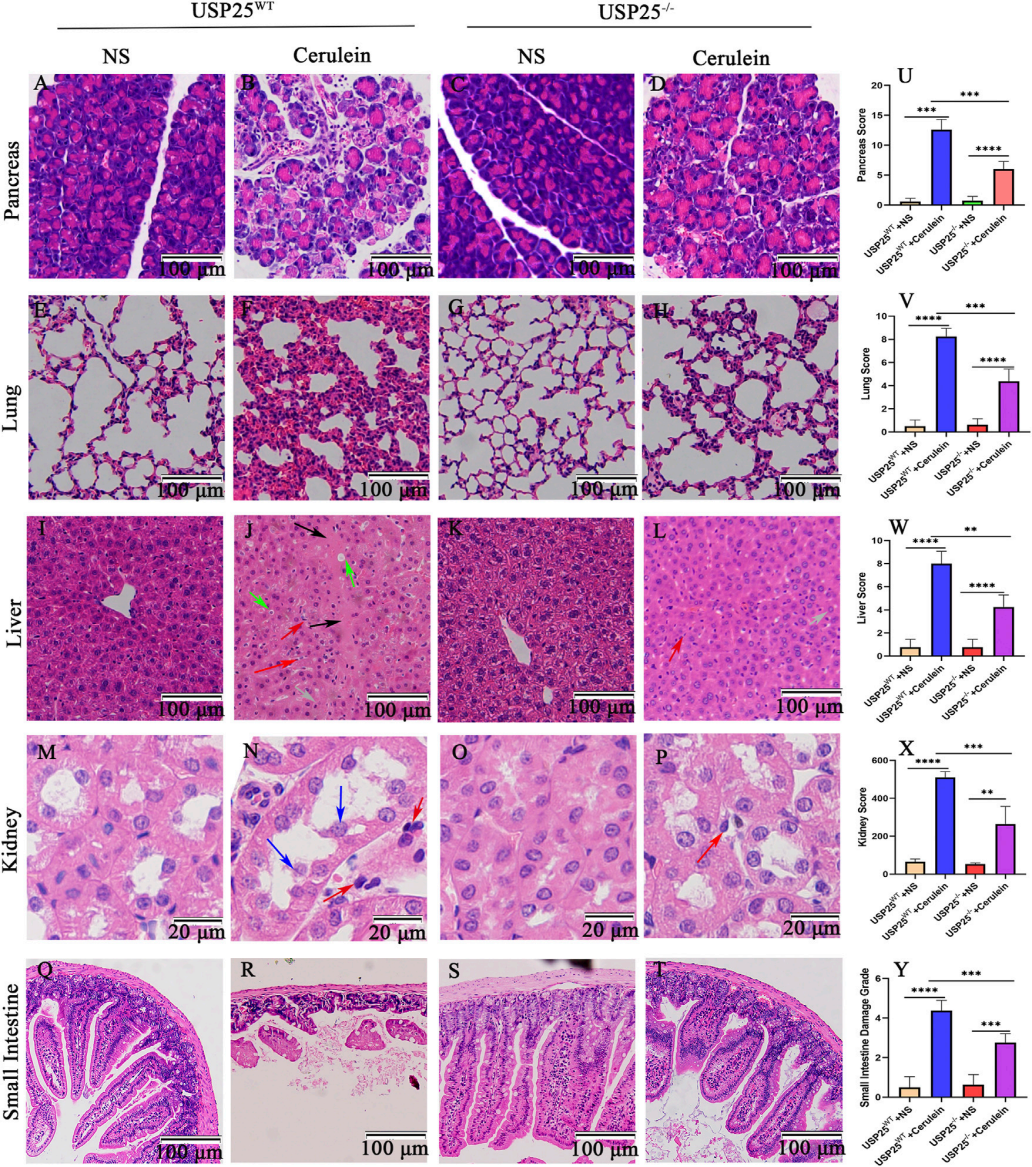
USP25 deficiency attenuates pancreatic inflammation and AP-related multiple organ injury *in vivo*. USP25^Wild−Type (WT)^ mice and USP25 knockout (USP25^−/−^) mice were randomly divided into four groups (n = 8 per group): USP25^WT^ + NS, USP25^WT^ + cerulein, USP25^−/−^ + NS, USP25^−/−^ + cerulein. **(A–T)** H&E staining of pancreas, lung, liver, kidney, and small intestine in four experimental groups (Black arrow: necrosis; red arrow: inflammatory cell; green arrow: cytoplasmic vacuolation; grey arrow: edema; blue arrow: detachment of tubular epithelial cell). **(U–Y)** The histological scores of pancreas, lung, liver, kidney, and small intestine in four experimental groups. **p < 0.01, ***p < 0.001 and ****p < 0.0001.

To study the contribution of USP25 in the pathogenesis of AP, we examined cerulein-induced AP in USP25 knockout (USP25^−/−^) mice. The DNA identification results of the experimental mice are shown in Supplementary Figure 2D. As shown in Figure 3, compared with USP25^WT^ mice, USP25 deficiency had a protective effect on cerulein-induced AP mice, which showed a lower degree of pancreatic inflammation and multiple organ injury. The relative weight of the pancreas of mice in the USP25^−/−^ group was significantly lower than that of the USP25^WT^ group (Supplementary Figure 2E). The damage to the pancreas, lung, liver, kidney, and small intestine were significantly lower in USP25^−/−^ mice than in USP25^WT^ mice, and the degree of edema, necrosis, inflammatory cell infiltration, and hemorrhage of the pancreas of USP25^−/−^AP mice were significantly lower than those of USP25^WT^ AP mice (*p* < 0.05). The lungs of USP25^−/−^AP mice showed less bleeding and interstitial and alveolar edema, inflammation, and cell infiltration than that of the USP25^WT^ AP mice (*p* < 0.05). In addition, hepatocellular edema, cytoplasmic vacuolation, necrosis, loss of intercellular boundaries and inflammatory cell infiltration in the livers of the USP25^−/−^AP mice decreased significantly (*p* < 0.05). Furthermore, dilated renal tubules, disordered tubular structure and inflammatory cell infiltration around the renal tubules were rarely observed in the kidneys of the USP25^−/−^AP mice (*p* < 0.05). Moreover, the villi tip and lamina propria were rarely peeled off or fell off in pieces in the small intestine of the USP25^−/−^AP mice (*p* < 0.05).

**FIGURE 4:**
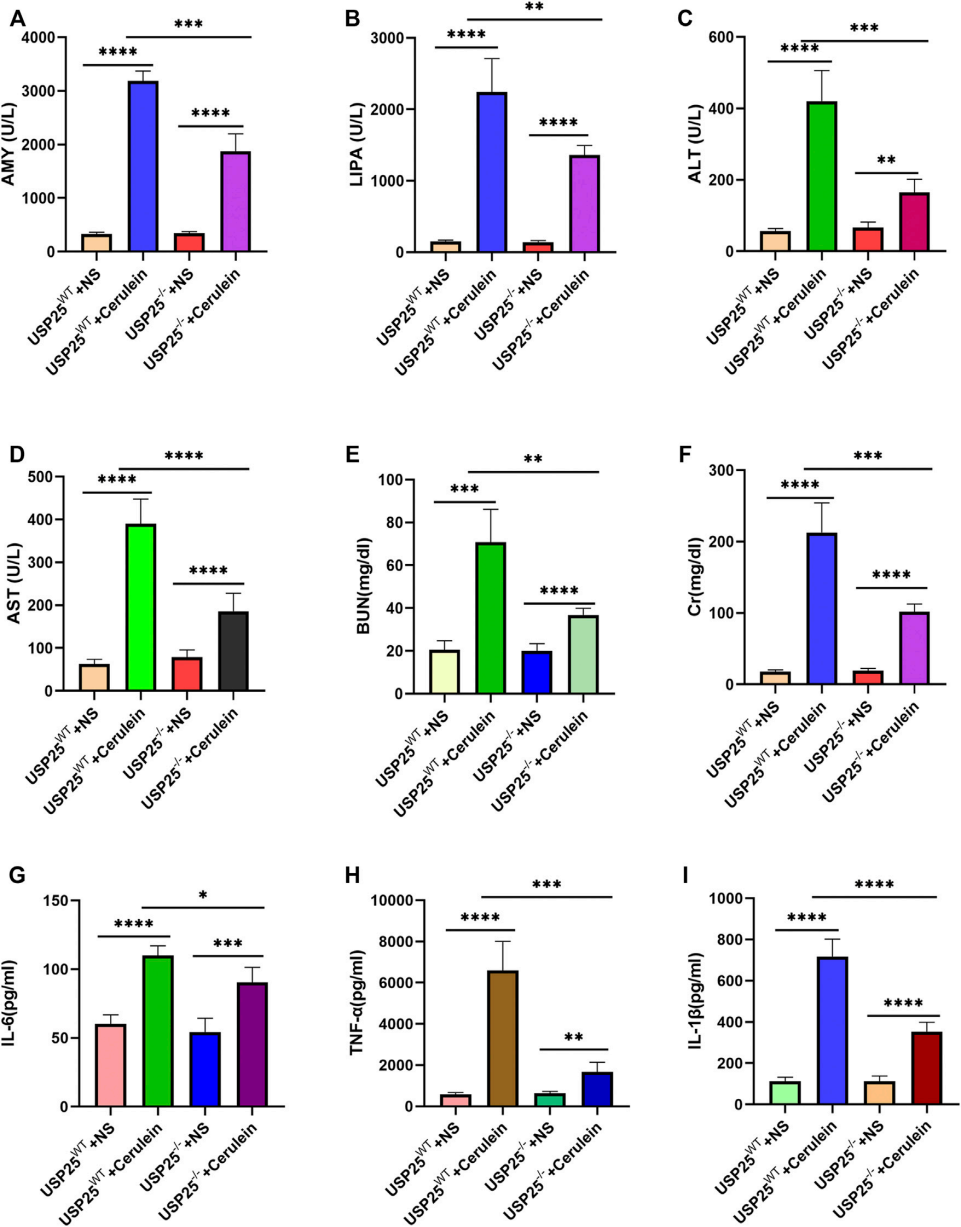
USP25 deficiency reduces mice serum indicators levels of acute pancreatitis and relieves acute pancreatitis-related multiple organ failure *in vivo*. **(A–F)** Mice serum AMY, LIPA, ALT, AST, Cr, BUN, CRP were examined by automatic chemical analyzer in four experimental groups (n = 8 per group), including USP25^WT^ + NS, USP25^WT^ + cerulein, USP25^−/−^ + NS, USP25^−/−^ + cerulein. **(G–I)** The levels of serum IL-6, TNF-α, and IL-1β in USP25^WT^ + NS, USP25^WT^ + cerulein, USP25^−/−^ + NS, USP25^−/−^ + cerulein mice were measured by ELISA (n = 8 per group). *p < 0.05, **p < 0.01, ***p < 0.001 and ****p < 0.0001.

Consistent with the above mentioned histology, the levels of serum AMY (*p* < 0.05), LIPA (*p* < 0.05), ALT (*p* < 0.05), AST (*p* < 0.05), BUN (*p* < 0.05), and Cr (*p* < 0.05) of the USP25^−/−^AP group were significantly lower than those of the USP25^WT^ AP group (Figures 4A–F). The results of serum IL-6, TNF-α, and IL-1β levels in mice are shown in Figures 4G–I. The levels of serum IL-6 (*p* < 0.05), TNF-α (*p* < 0.05), and IL-1β (*p* < 0.05) in the USP25^−/−^AP group were also significantly lower than those in the USP25^WT^ AP group.

These experimental results suggest that USP25 aggravates AP and AP-related multiple organ injury.

### USP25 Aggravates AP by Destroying Tight Junctions of the Pancreas

**FIGURE 5:**
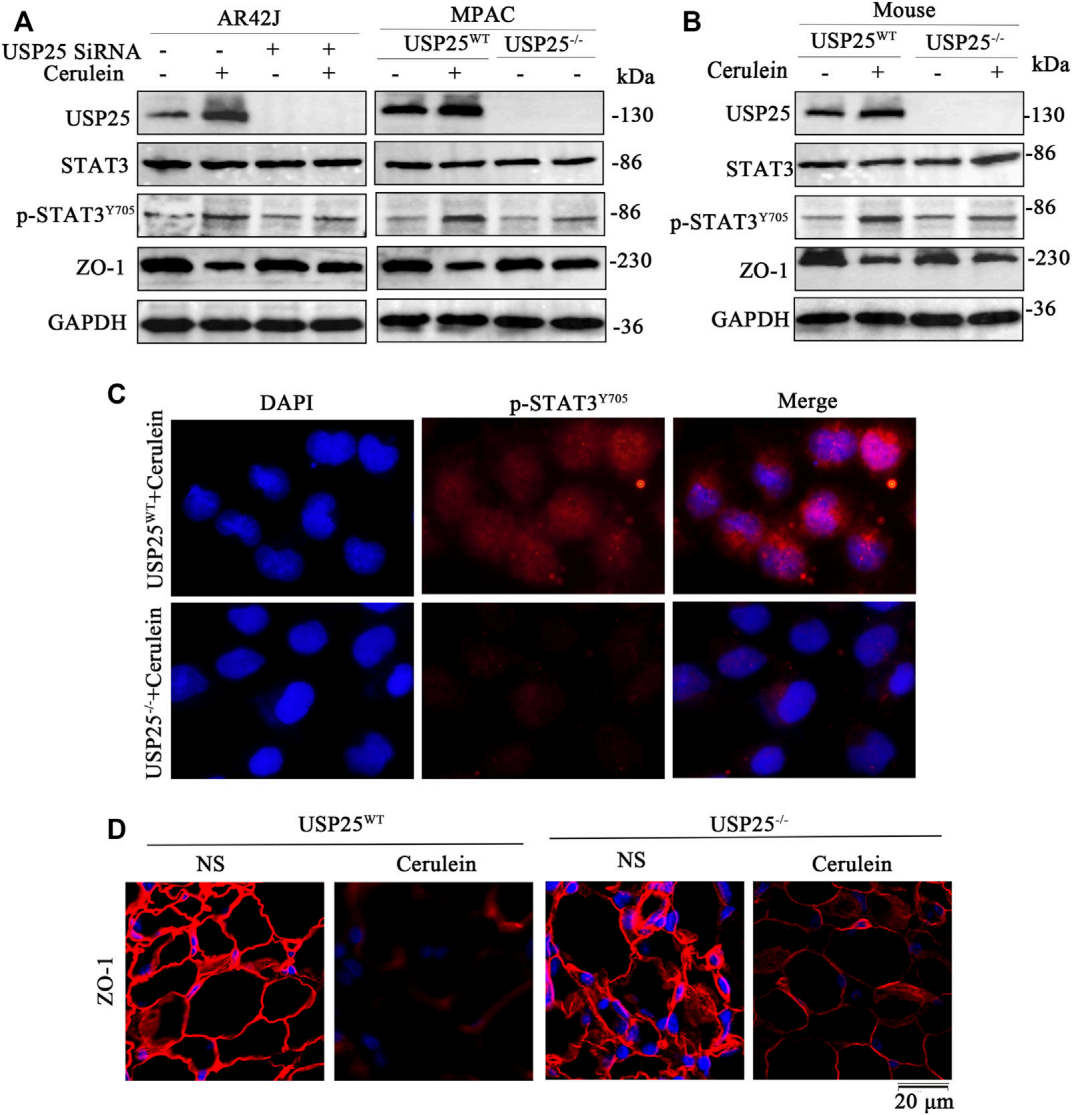
USP25 exacerbates acute pancreatitis by destroying tight junctions of the pancreas and STAT3 activation may be involved *in vitro* and *in vivo*. **(A)** Western blotting was used to measure the protein levels of USP25, STAT3, p-STAT3Y705 and ZO-1 protein in AR42J, MPAC cells transferred with nontargeting SiRNAs (NC) or USP25 SiRNA and induced AP with cerulein. **(B)** Western blotting was performed to measure the protein levels of USP25, STAT3, p-STAT3Y705, and ZO-1 in mice pancreases of the following four groups: USP25WT + NS, USP25WT + cerulein, USP25^−/−^ + NS, USP25^−/−^ + cerulein **(C)** The expression level of p-STAT3Y705 were examined by immunofluorescence in MPAC of two groups: USP25WT + cerulein and USP25^−/−^ + cerulein **(D)** ZO-1 were examined by immunofluorescence in mice pancreases of four groups: USP25WT + NS, USP25WT + cerulein, USP25^−/−^ + NS, USP25^−/−^ + cerulein mice.

A study by Fallon et al. showed that in the early stage of AP, the structure and function of the paracellular barrier in the acinar and lobules are destroyed, which may be related to early clinical features (Fallon et al., 1995). The expression of ZO-1, an important tight junction protein, decreases significantly in AP. We used IF and WB to detect the expression of tight junction proteins in mouse pancreases and the results showed that the level of ZO-1 proteins was significantly reduced (IF, *p* < 0.05; WB, *p* < 0.05) (Figures 2C,D, Supplementary Figures 2F, G). In addition, both *in vivo* and *in vitro* experiments confirmed that the damage of tight junctions in the USP25 deficiency AP group was significantly less than that in the USP25^WT^ AP group (Figures 5A,B,D, Supplementary Figures 3D, 3H, 3L, 3N).

### Therefore, USP25 Exacerbates AP by Destroying Tight Junctions of the Pancreas

#### USP25 Aggravates AP and AP-Related Multiple Organ Injury by Destroying Tight Junctions Through Activation of the STAT3 Pathway

To explore the possible mechanisms of USP25 in AP and AP-related multiple organ injury, we used USP25 siRNA and cerulein to intervene in AR42J. Cell protein was extracted to verify transfection efficiency (Supplementary Figures 2H, 2I), moreover, MPAC were isolated from 8-week-old male USP25 KO and WT mice, and cerulein was used to induce AP in MPAC, and then the levels of USP25, STAT3, p-STAT3 (Try705), and ZO-1 protein were examined. As shown in Figure 5A and Supplementary Figures 3A–3H, the level of p-STAT3 protein in the USP25 siRNA + cerulein group or USP25^−/−^ + cerulein was significantly lower than that in the NC + cerulein group or USP25^WT^ + cerulein, while the ZO-1 protein level in siRNA + cerulein group or USP25^−/−^ + cerulein was significantly higher than in NC + cerulein group or USP25^WT^ + cerulein. Similarly, as shown in Figure 5B and Supplementary Figures 3I–3L, in cerulein-induced AP mice, the level of p-STAT3 protein in USP25^−/−^ AP mice was significantly lower than that in USP25^WT^ AP group, but ZO-1 in USP25^−/−^ AP mice was significantly higher than in USP25^WT^ AP mice. Expression levels p-STAT3 in MPAC and ZO-1 levels in pancreatic tissues respectively measured using IF were consistent with the results of the WB (Figures 5C,D and Supplementary Figures 3M–3N). These results suggest that USP25 may aggravate AP-and AP-related multiple organ injury by promoting STAT3 phosphorylation.

**FIGURE 6:**
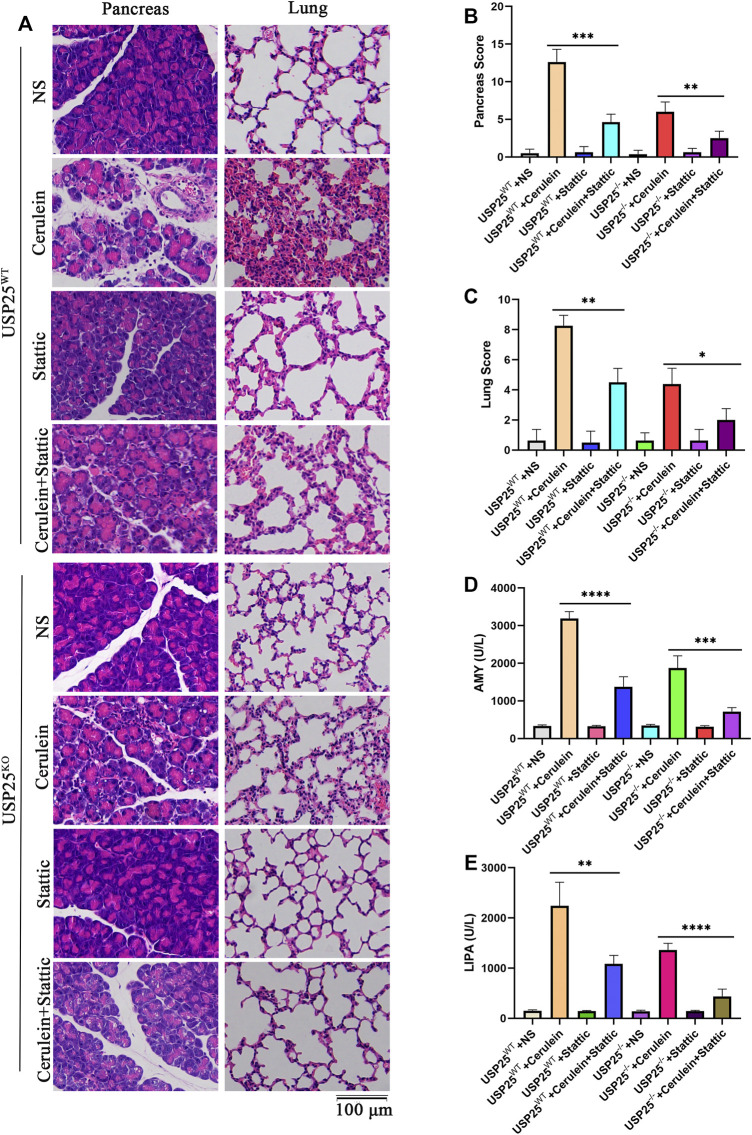
USP25 aggravates acute pancreatitis and acute pancreatitis-related lung injury by activating STAT3 pathway *in vivo*. STAT3 (Y705) activation inhibitor-Stattic was used to interfere with STAT3 phosphorylation in USP25^WT^ and USP25^−/−^mice (n = 8 per group), then cerulein was used to induce AP in mice. **(A)** H&E staining of pancreas, lung in eight experimental groups. **(B–C)** The histological scores of pancreas, lung in eight experimental groups. **(D–E)** Mice serum AMY, LIPA were examined by automatic chemical analyzer in eight experimental groups. *P<0.05, **P<0.01, ***p<0.001 and ****p<0.0001.

**FIGURE 7:**
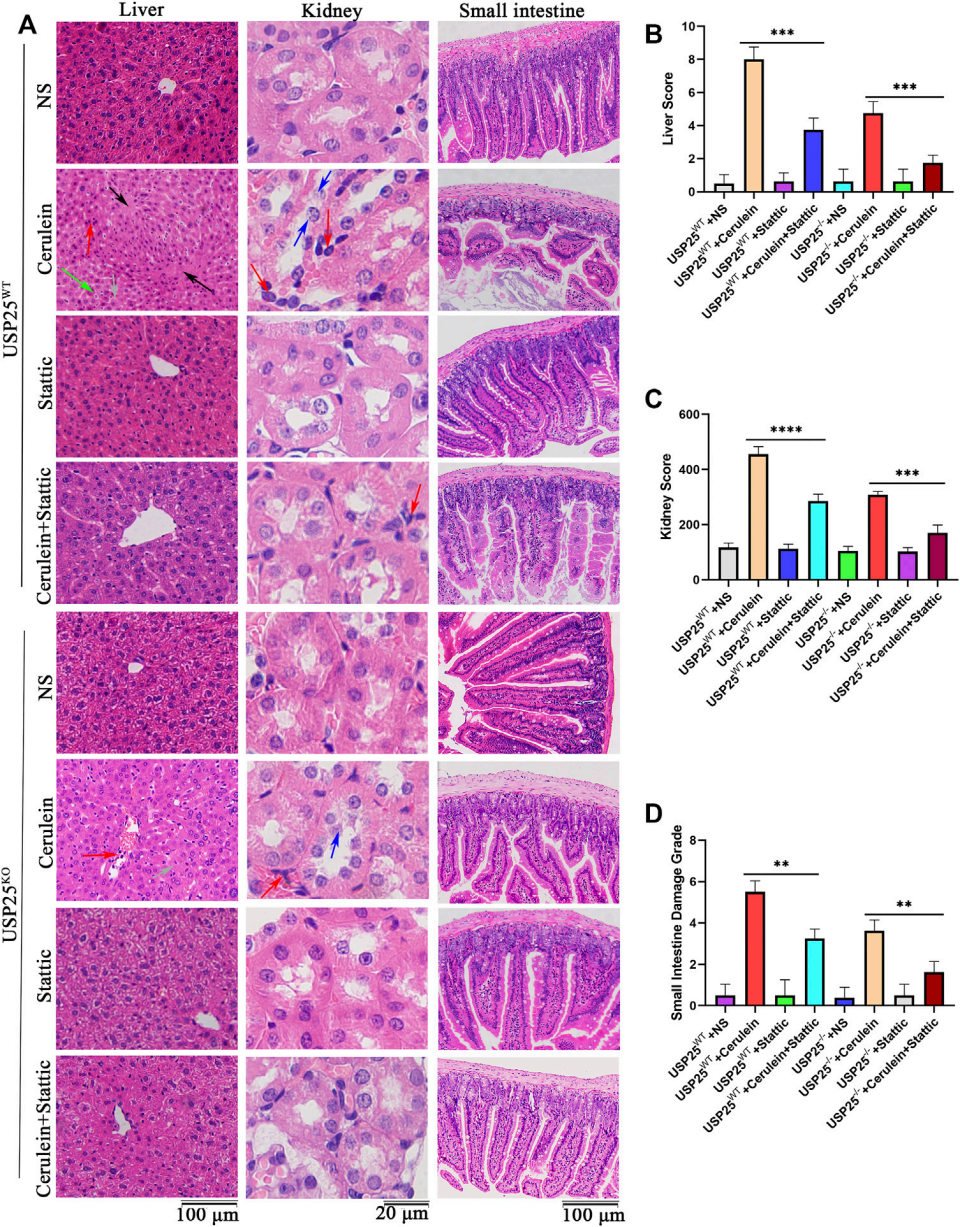
USP25 aggravates acute pancreatitis-related liver, kidney, and small intestine injury through activating STAT3 pathway *in vivo*. Stattic was used to interfere with STAT3 phosphorylation in USP25^WT^ and USP25^−/−^mice, then cerulein was used to induce AP in mice. **(A)** H&E staining of liver, kidney and small intestine in eight experimental groups (Black arrow: necrosis; red arrow: inflammatory cell; green arrow: cytoplasmic vacuolation; grey arrow: edema; blue arrow: detachment of tubular epithelial cell). **(B–D)** The histological scores of liver, kidney and small intestine in eight experimental groups. **P<0.01, ***p<0.001 and ****p<0.0001.

To verify the above hypothesis, we used the STAT3 (Y705) activation inhibitor stattic to interfere with STAT3 phosphorylation in mice and the cells AR42J and MPAC. The results showed that the relative weight of the pancreas in AP group with stattic was significantly lower than that in AP group without stattic (*p* < 0.05) (Supplementary Figure 4A). H&E staining in various organs of mice (Figures 6, 7) showed less inflammatory damage in the AP group with stattic than in the AP group without stattic: pancreas (*p* < 0.05), lung (*p* < 0.05), liver (*p* < 0.05), kidney (*p* < 0.05), and small intestine (*p* < 0.05). In addition, the levels of AMY (*p* < 0.05) and LIPA (*p* < 0.05) in the AP group with stattic were significantly lower than those in the AP group without stattic (Figures 6D,E).

**FIGURE 8:**
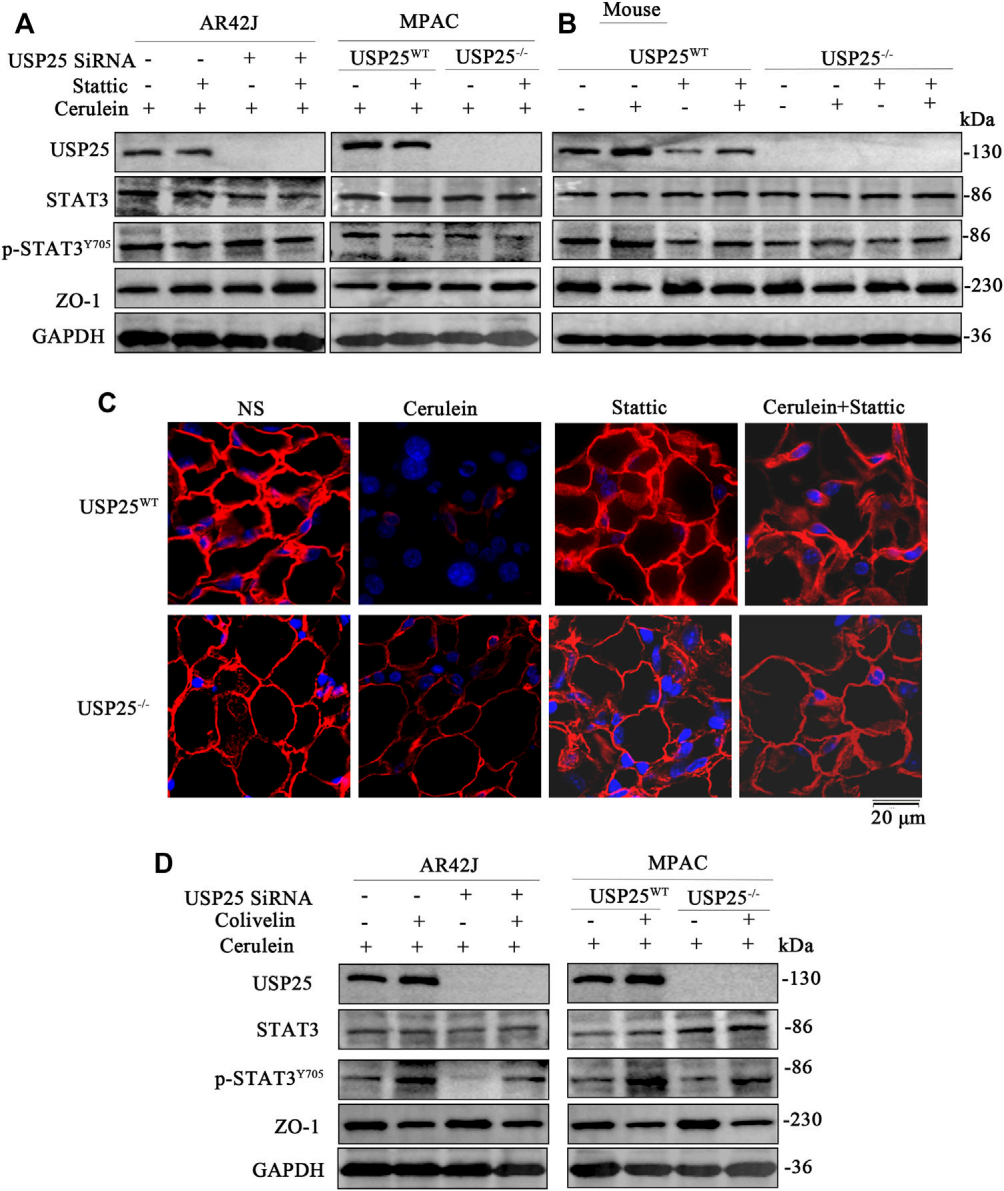
USP25 aggravates acute pancreatitis and acute pancreatitis-related multiple organ injury by destroying tight junctions through activating STAT3 pathway *in vitro* and *in vivo*. Stattic was used to interfere with STAT3 phosphorylation in AR42J, MPAC cells and mice (n = 8 per group), then cerulein was used to induce AP. Colivelin (STAT3 agonist) was used to activate STAT3 in AR42J and MPAC cells, and then cerulein was used to induce AP in cells **(A–B)** Western blotting was performed to measure the protein levels of USP25, STAT3, p-STAT3^Y705^, and ZO-1 in AR42J, MPAC cells and mice pancreases after stattic treatment. **(C)** Immunofluorescence was performed to measure the protein level of ZO-1 in mice pancreases of eight groups. **(D)** Western blotting was performed to measure the protein levels of USP25, STAT3, p-STAT3^Y705^, and ZO-1 in AR42J, MPAC cells after colivelin treatmenet.

Furthermore, WB was used to examine the protein expression levels in the AR42J, MPAC, and mouse pancreatic tissue. The results are shown in Figures 8A,B, Supplementary Figures 4B–4M, where the levels of USP25 protein (*p* < 0.05), p-STAT3 (*p* < 0.05) of AP group with stattic were significantly reduced compared to those in AP group without stattic. In contrast, the expression of ZO-1 protein in AP group with stattic was significantly higher (*p* < 0.05) than in AP group without stattic. We used the IF method to examine the expression level of ZO-1 in the pancreatic tissue (*p* < 0.05), which was consistent with the results of WB (Figure 8C and Supplementary Figure 4N).

Consistent with the above mentioned results, ELISA results of serum inflammatory factors showed that compared with the AP group without stattic, the serum IL-6 (*p* < 0.05), TNF-α (*p* < 0.05), and IL-1β (*p* < 0.05) in the AP group with stattic were significantly reduced, suggesting that inflammation of pancreas in AP group was reduced (Supplementary Figures 5A–5C).

To further verify that USP25 regulates STAT3, the STAT3 agonist colivelin was added to AR42J and MPAC, and then cerulein was used to induce AP in the cells. As shown in Figure 8D and Supplementary Figures 5D–5K, after the addition of colivelin to cells, the expression of p-STAT3 (Y705) increased (*p* < 0.05), while the expression of ZO-1 decreased significantly (*p* < 0.05), which suggest that activation of STAT3 by colivelin counteracted the protective effect of USP25 deficiency on AP in AR42J, and MPAC.

Therefore, USP25 aggravates AP-and AP-related multiple organ injury by destroying tight junctions through activating the STAT3 pathway.

## Discussion

Patients with AP can experience systemic damage caused by a systemic inflammatory response, which manifests as multiple organ injuries. The proportion of AP patients with organ failure is 8–20% (Hamada et al., 2014). In tertiary health care hospitals with many AP patients, the proportion of SAP patients may be as high as 40% (Schepers et al., 2019). The inflammatory response often involves the lungs, heart, liver, and kidney, which seriously affects the prognosis of patients. In our study, 22.78% of patients were diagnosed with SAP, the proportion of patients with SAP was consistent with previous studies.

Inflammation is a local and systemic pathological reaction in AP. During the development of AP, pancreatic acinar cells release digestive enzymes, inflammatory cytokines, and pro-inflammatory mediators into the blood circulation, including IL-6, TNF-α, and IL-1β (Habtezion et al., 2019). Pro-inflammatory mediators enter the liver through the portal vein, leading to the activation and proliferation of intrinsic Kupffer cells, which in turn produce more pro-inflammatory cytokines, inducing early liver injury and the release of hepatocyte pro-inflammatory factors (Wang et al., 2017). Liver-derived cytokines activate alveolar macrophages through the intravenous route to release chemotactic cytokines to recruit neutrophils. Neutrophils and elevated circulating pro-inflammatory cytokines severely damage the alveolar epithelium, leading to pulmonary edema, and then produce inflammatory cytokines that cause systemic inflammatory response syndrome (Yang et al., 2016). Inappropriate inflammatory signal activation and systemic inflammatory response syndrome are powerful triggers for local kidney inflammation and subsequent tissue damage (Lee et al., 2019).

In this study, it was observed that serum AMY, LIPA, etc., and pro-inflammatory cytokines (IL-6, TNF-α, IL-1β) increased after cerulein injection, and the pathological damage to the pancreas and other organs (including liver, lung, kidney, and small intestine) were aggravated. These results were consistent with the results of previous studies (Wen et al., 2020), indicating that cerulein injection can successfully induce AP in mice.

Our results show that serum USP25 is highly expressed in patients with AP and is positively correlated with inflammation and organ injury. In the cerulein-induced AP mice study, it was further confirmed that the expression of USP25 was upregulated in AP mice. This conclusion is consistent with the following previous studies that have shown that USP25 plays an important role in immune regulation. Lipopolysaccharides and viral infections activate USP25 transcription through signals triggered by type I interferon (IFN). In addition, signals triggered by type I IFN transduction induce the expression and protein synthesis of IFN regulatory factor 7 (IRF7). IRF7 binds to the USP25 promoter to activate the transcription of the *Usp25* gene (Ren et al., 2016). USP25 can promote the intestinal inflammatory response during bacterial infection, and the goblet cells and Paneth cells in USP25^WT^ mice are significantly decreased compared with those in the USP25^−/−^ mice (Wang et al., 2020).

STAT3 activation plays an important role in the development of AP. Research by Li et al. found that when mouse AP was caused by intraductal hypertension, STAT3 phosphorylation was activated, serum AMY, IL-6, TNF-α, and IL-1β levels were all significantly increased, and the tight junction of the pancreas disappeared (the expression levels of ZO-1 and occludin decreased) (Wen et al., 2018). In addition, studies have shown that activation of STAT3 aggravates AP-related liver damage in cerulein-induced AP in mice (Ren et al., 2021), which is consistent with our study result that STAT3 activation in pancreatitis leads to damage to tight junctions.

USP25, as a member of the deubiquitination family, can effectively reduces protein degradation by the proteasome through reversibly removing the ubiquitin chain. USP25 may regulate the inflammatory response in AP through multiple signaling pathways. Nino et al. found that USP25 restrained the degradation of the EGFR by assisting in the association of the E3 ubiquitin ligase c-Cbl with EGFR, thereby modulating the amplitude of ubiquitylation on the receptor. (Nino et al., 2020). Previously, Ardito et al. demonstrated that EGFR was required for caerulein-induced pancreatitis and kras-induced tumorigenesis (Ardito et al., 2012). Moreover, Zhong et al. identified USP25 as a regulator of TLR signaling, USP25 was recruited to the TLR4 signaling complex, and specifically reversed the Lys48-linked ubiquitination of TRAF3 that was mediated by the E3 ubiquitin ligase cIAP2 (cellular inhibitor of apoptosis 2) (Zhong et al., 2013). Previously, Sharif et al. demonstrated that TLR4 played a significant pro-inflammatory role in the progression of acute pancreatitis (Sharif et al., 2009). Furthermore, when mouse colitis is associated with bacterial infection, USP25 can aggravate colitis by promoting STAT3 phosphorylation (Wang et al., 2020), consistent with previous studies, our study found that USP25 aggravates AP by regulating STAT3 (especially p-STAT3). We speculate that USP25 regulates AP by removing the ubiquitin chain of p-STAT3 to reduce the ubiquitination and degradation of p-STAT3. The increase of p-STAT3 activates the inflammatory signaling pathway, thereby increasing the release of inflammatory factors, which damages tight junctions and aggravates acute pancreatitis. However, this speculation needs to be further confirmed by experiments.

## Conclusion

Our current work demonstrates the role of USP25 in AP and AP-related multiple organ injuries. First, USP25 is highly expressed in patients with AP and is positively correlated with the degree of inflammation in AP and AP-related multiple organ injury. Second, we confirmed that USP25 deficiency can alleviate cerulein-induced AP and AP-related multiple organ injury. Last, our results show that USP25 promotes the release of pro-inflammatory factors and destroys the tight junction of the pancreas by activating the STAT3 pathway, thereby aggravating AP. Therefore, our conclusion emphasizes the role of USP25 in exacerbating AP-and AP-related multiple organ injuries. Targeting the action of USP25 may be a potential therapeutic option for treating AP.

## Data Availability

The raw data supporting the conclusions of this article will be made available by the authors, without undue reservation.

## Supplementary Material

Supplementary Figure S1 Cerulein induces acute pancreatitis and acute pancreatitis -related multiple organ injury in mice. Wide type mice were randomly divided into 2 groups, control group: USP25^WT^+ normal saline (NS), AP group: USP25^WT^+ cerulein. **(A)** Tissues of pancreas, lung, liver, kidney, and small intestine from two groups were stained for CD45. **(B–F)** The number of CD45 positive cells in organs from mice treated with saline or cerulein were quantified.

Supplementary Figure S2 The expression of USP25 is up-regulated in cerulein-induced acute pancreatitis mice and USP25 deficiency attenuates pancreatic inflammation. Wide type mice (WT) were randomly divided into 2 groups, control group: USP25^WT^+ normal saline (NS), AP group: USP25^WT^+ cerulein. Besides, USP25 knock-out mice were also randomly divided into 2 groups, control group: USP25^−/−^+ normal saline (NS), AP group: USP25^−/−^+ cerulein. **(A)** The relative weight (the mouse pancreas weight (g)/mouse weight (g) of the pancreas in two WT groups. **(B)** The expression level of USP25 protein in two WT groups examined by immunohistochemistry were quantified. **(C)** The protein expression of USP25 mesured by Western blotting in two WT groups were quantified. **(D)** A 2% agarose gel electrophoresis was used to verify the DNA of USP25WT and USP25^−/−^ mice. **(E)** The relative weight (the mouse pancreas weight (g)/mouse weight (g) )of the pancreas in four mice groups (USP25WT+ NS, USP25WT+cerulein, USP25^−/−^+NS, USP25^−/−^+ cerulein) were compared. **(F)** The protein expression of ZO-1 mesured by immunofluorescence were quantified in two WT groups. **(G)** The protein expression of ZO-1 mesured by Western blotting in two WT groups were quantified. **(H, I)** The protein expression of USP25 in AR42J cells cocultured with PBS (Control), nontargeting SiRNA (NC), Si001, Si002 or Si003 were examined by Western blotting and quantified respectively. *p<0.05, **P<0.01, ***p<0.001 and ****p<0.0001.

Supplementary Figure S3 USP25 deficiency attenuates pancreatic inflammation and acute pancreatitis -related multiple organ injury *in vitro* and *in vivo.* **(A–L)** The protein expression of USP25, STAT3, p-STAT3^Y705^ and ZO-1 in AR42J, MPAC (NC+PBS, NC+cerulein, USP25 SiRNA+PBS, USP25 SiRNA+ cerulein) and mice (USP25^WT^+ NS, USP25^WT^+cerulein, USP25^−/−^+NS, USP25^−/−^+ cerulein) in four groups mesured by Western blotting were quantified. **(M)** The protein expression of p-STAT3^Y705^ mesured by and immunofluorescence were quantified in MPAC cells of two groups:USP25^WT^+cerulein, USP25^−/−^+ cerulein. **(N)** The protein expression of ZO-1 mesured by immunofluorescence were quantified in four mice groups: USP25WT+ NS, USP25WT+cerulein, USP25^−/−^+NS, USP25^−/−^+ cerulein.The expression level of ZO-1 protein in two groups examined by immunofluorescence were quantified. *p<0.05, **P<0.01, ***p<0.001 and ****p<0.0001.

Supplementary Figure S4 USP25 aggravates acute pancreatitis and acute pancreatitis-related multiple organ injury by destroying tight junctions through activating STAT3 pathway. Stattic was used to interfere with STAT3 phosphorylation in AR42J cells (nontargeting SiRNA(NC) and USP25 SiRNA cells) and mice (USP25^WT^ and USP25^−/−^mice), then cerulein was used to induce AP. **(A)** The relative weight (the mouse pancreas weight (g)/mouse weight (g) of the pancreas in eight groups were compared. **(B–M)** The protein expression of USP25, STAT3, p-STAT3Y705 and ZO-1 measured by Western blotting in AR42J, MPAC cells and tissues from mice of eight groups were quantified. **(N)** The expression levels of ZO-1 protein in eight groups examined by immunofluorescence were quantified. *p<0.05, **P<0.01, ***p<0.001 and ****p<0.0001.

Supplementary Figure S5 USP25 aggravates acute pancreatitis and acute pancreatitis-related multiple organ injury by destroying tight junctions through activating STAT3 pathway. Stattic was used to interfere with STAT3 phosphorylation in mice, then cerulein was used to induce AP. Moreover, Colivelin (STAT3 agonist) was used to activate STAT3 in AR42J (nontargeting SiRNA and USP25 SiRNA cells) and MPAC cells, and then cerulein was used to induce AP in cells. **(A–C)** The levels of serum inflammatory factor IL-6, TNF-α, and IL-1β in eight groups mice after stattic treatment were measured by ELISA. **(D–K)** The protein expression of USP25, STAT3, p-STAT3Y705 and ZO-1 measured by Western blotting in AR42J and MPAC cells were quantified. **P<0.01, ***p<0.001 and ****p<0.0001.

## Abbreviations

AMY, amylase; AP, acute pancreatitis; ALT, alanine amiotransferase; AST, aspartate amiotransferase; BUN, blood urea nitrogen; CRP, C-creative protein; Cr, creatinine; ELISA, enzyme linked immunosorbent assay kit; IHC, immunohistochemistry; IF, immunofluorescence; IL, interleukin; LIPA, lipase; MAP, mild acute pancreatitis; MSAP, moderate severe acute pancreatitis; SAP, severe acute pancreatitis; STAT3, signal transducer and activator of transcription 3; TNF, tumor necrosis factor; USP25, ubiquitin-specific protease 25.
