# Supplementary figures and images for: Ubiquitin-Specific Protease 25 Aggravates Acute Pancreatitis and Acute Pancreatitis-Related Multiple Organ Injury by Destroying Tight Junctions Through Activation of The STAT3 Pathway
Source: Front Cell Dev Biol. 2022 Jan 13;9:806850. doi: 10.3389/fcell.2021.806850 (PMC8793747; doi:10.3389/fcell.2021.806850)

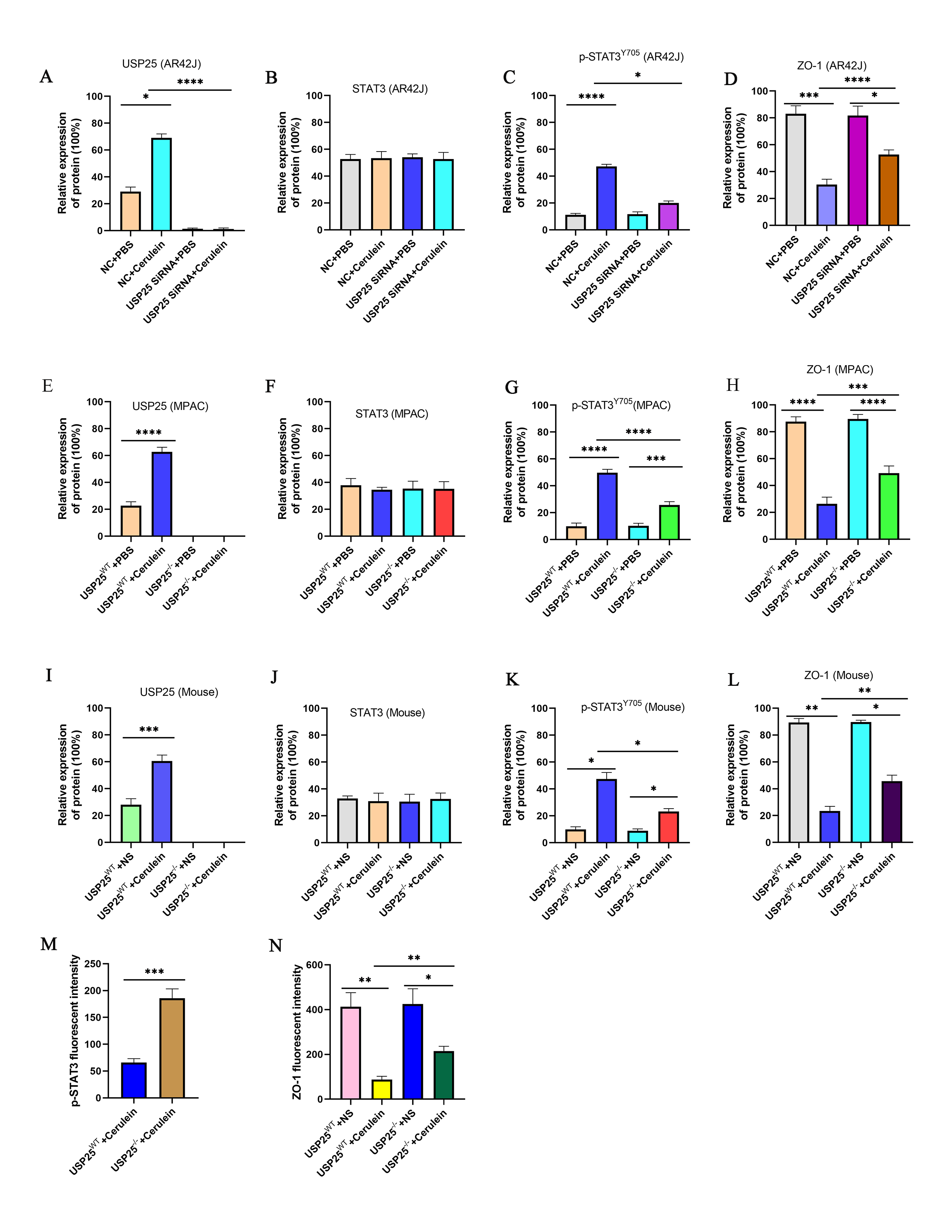

Supplement: Supplementary file 1 [file Image3.TIF]

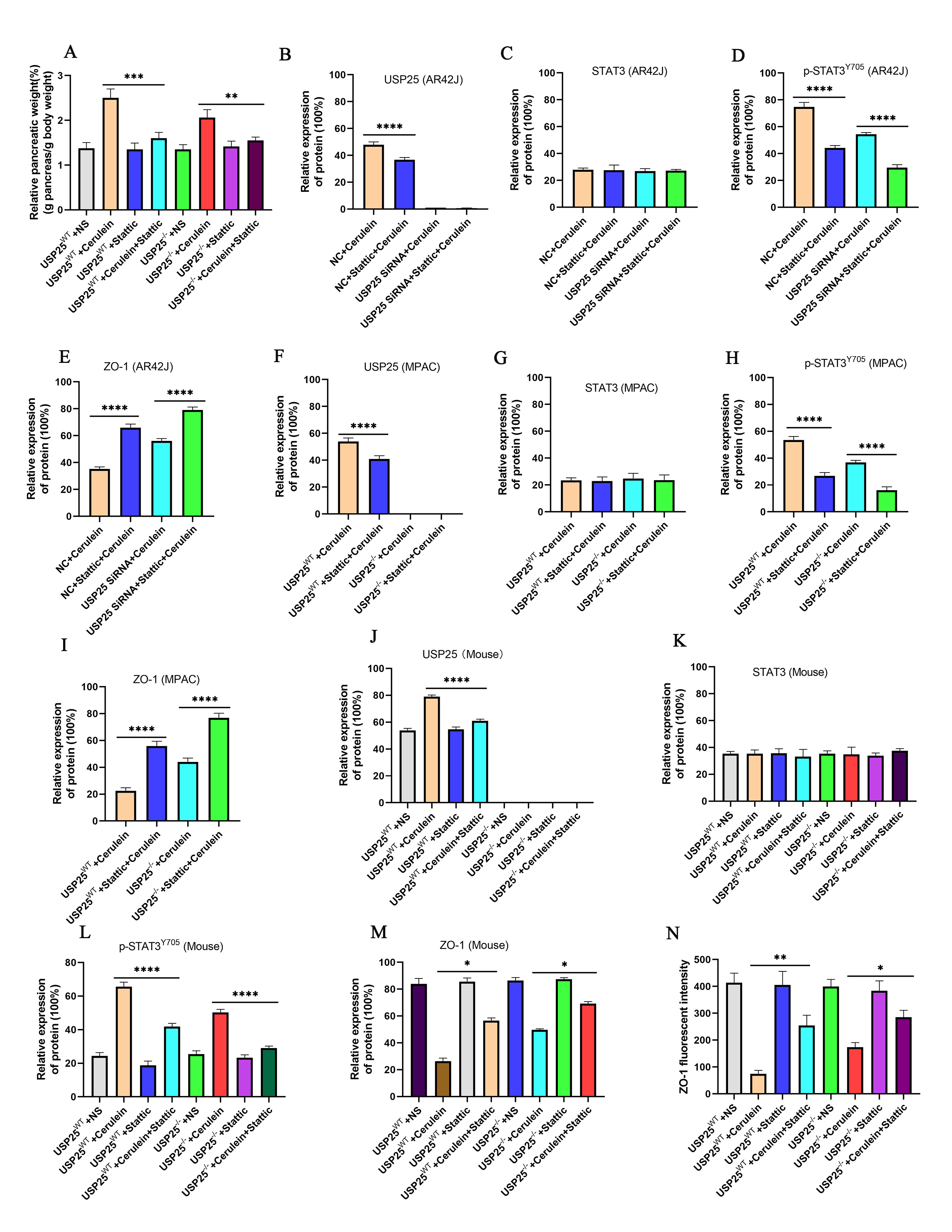

Supplement: Supplementary file 2 [file Image4.TIF]

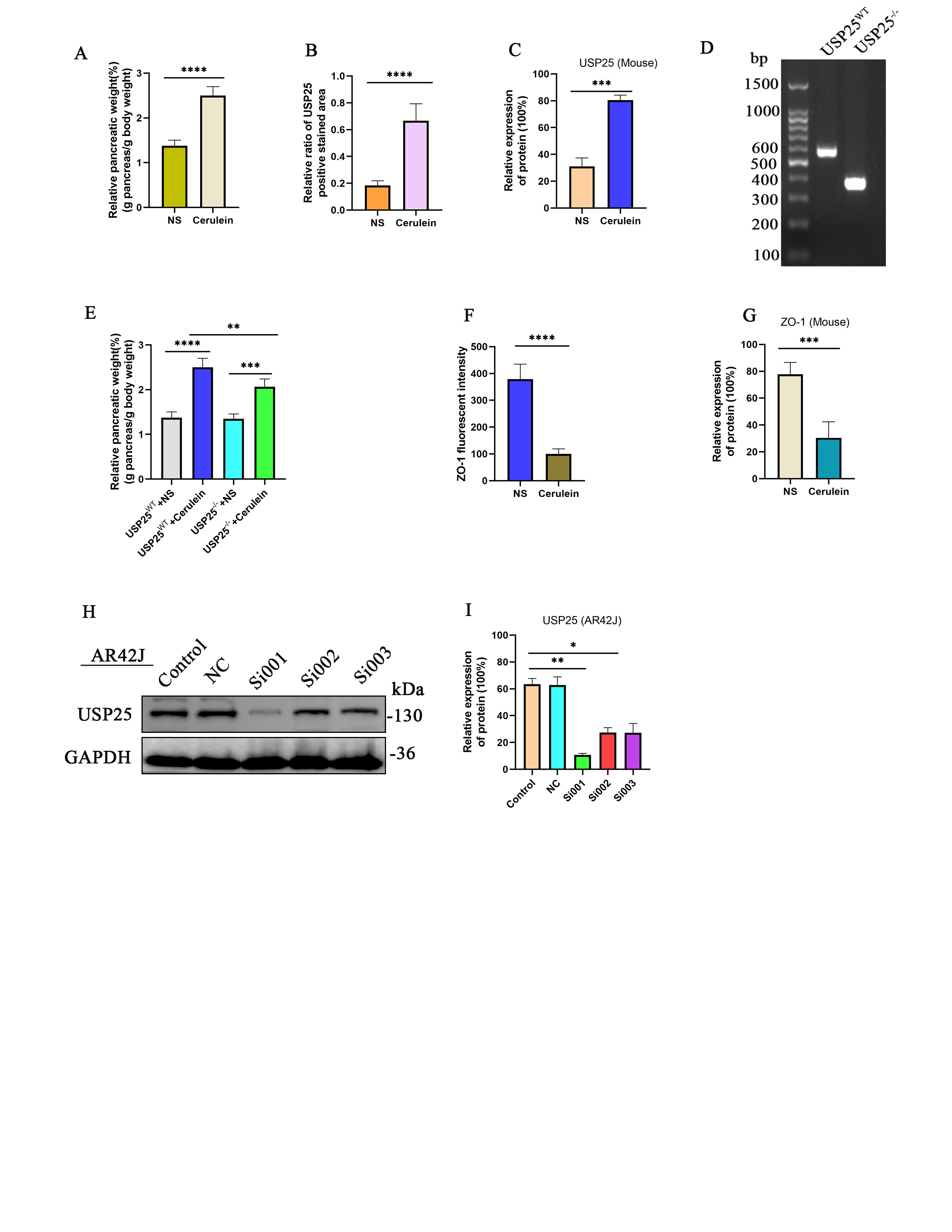

Supplement: Supplementary file 3 [file Image2.TIF]

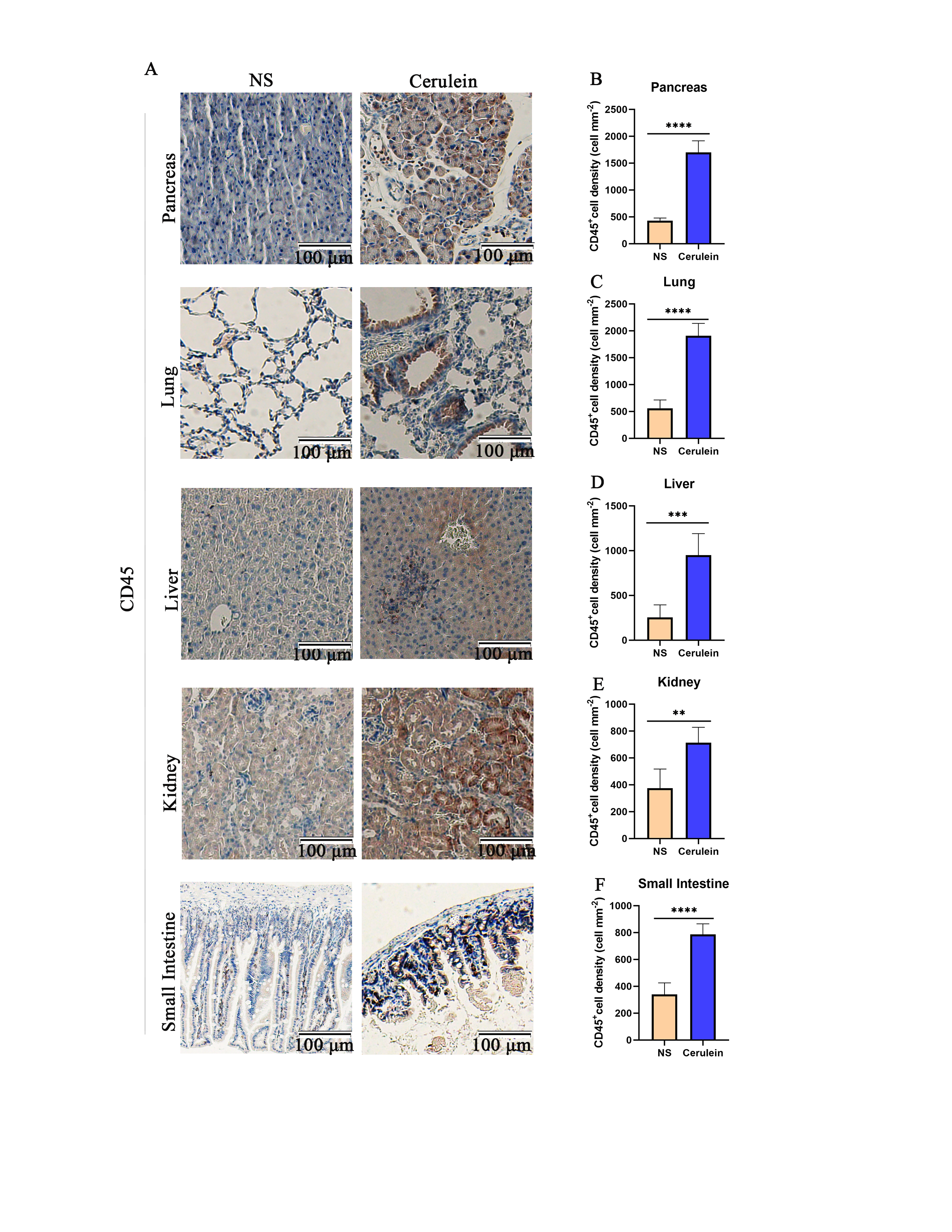

Supplement: Supplementary file 4 [file Image1.TIF]

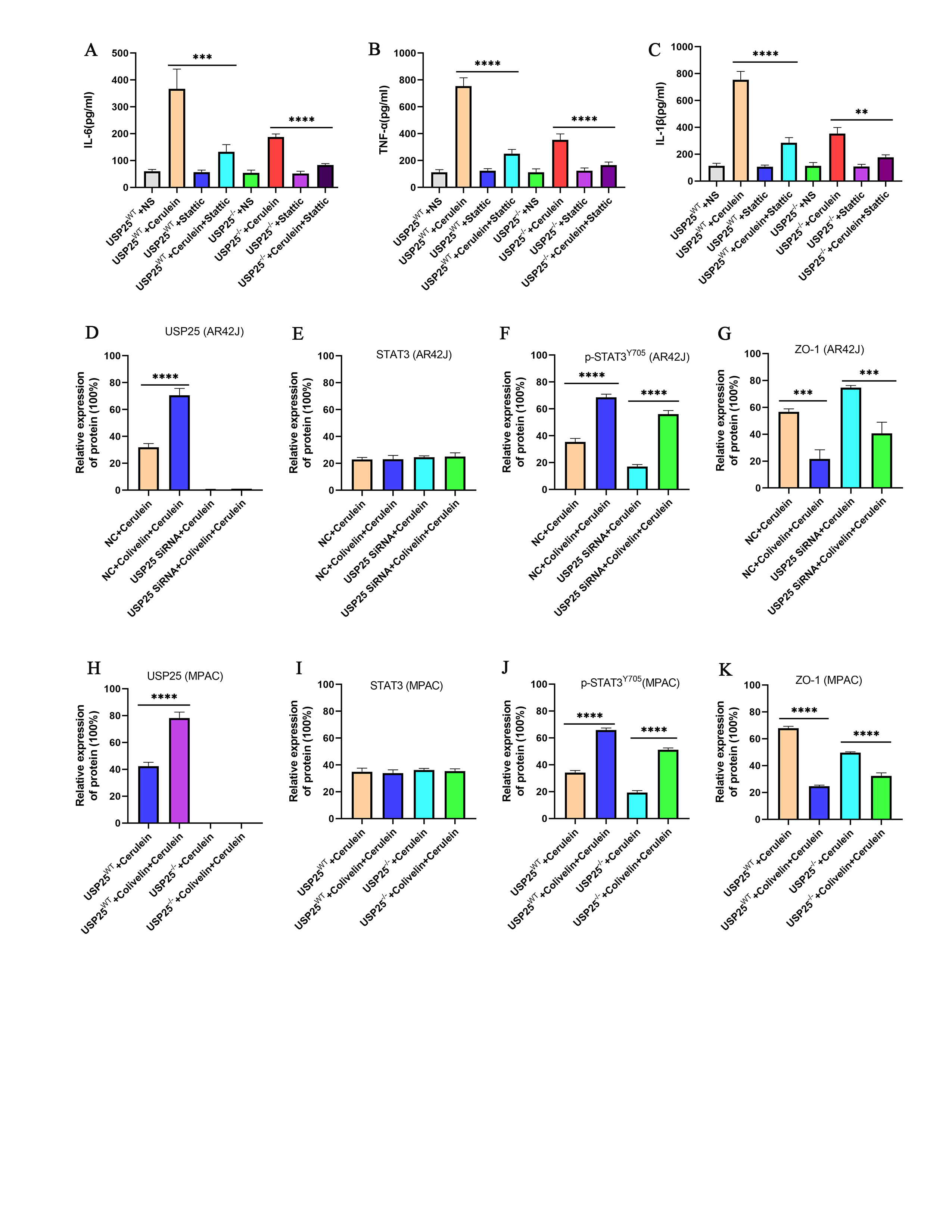

Supplement: Supplementary file 5 [file Image5.TIF]
